# 4,5-Dichloro-2*H*-1,3-oxazine-2,6(3*H*)-dione

**DOI:** 10.1107/S1600536809034606

**Published:** 2009-09-05

**Authors:** Damon Parrish, Brian Glass, Gretchen M. Rehberg, Margaret E. Kastner

**Affiliations:** a Department of Chemistry, Bucknell University, Lewisburg, PA 17837, USA

## Abstract

In the title compound, C_4_HCl_2_NO_3_, the essentially planar (maximum deviation = 0.023 Å for the ring O atom) mol­ecules form N—H⋯O hydrogen bonds between mol­ecules lying about inversion centers, forming eight-membered rings with an *R*_2_^2^(8) motif in graph-set notation.

## Related literature

For synthetic background, see: Warren *et al.* (1975 ▶); Rehberg & Glass (1995 ▶). For related structures, see: Copley *et al.* (2005 ▶); Parrish, Leuschner *et al.* (2009 ▶); Parrish, Tivitmahaisoon *et al.* (2009 ▶). For graph-set notation in hydrogen bonding, see: Bernstein *et al.* (1994 ▶).
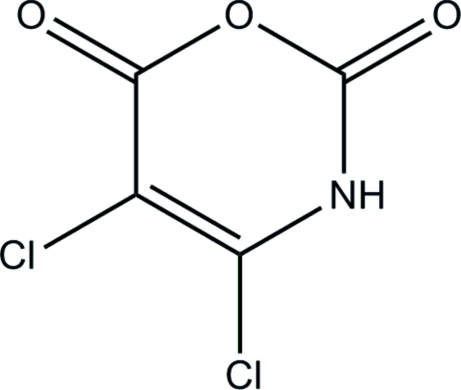


## Experimental

### 

#### Crystal data

C_4_HCl_2_NO_3_
*M* *_r_* = 181.96
Monoclinic, 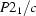

*a* = 10.2290 (16) Å
*b* = 5.2549 (8) Å
*c* = 12.2766 (16) Å
β = 112.359 (11)°
*V* = 610.28 (16) Å^3^
*Z* = 4
Mo *K*α radiation
μ = 1.00 mm^−1^
*T* = 293 K
0.38 × 0.33 × 0.15 mm

#### Data collection

Siemens R3m/V diffractometer
Absorption correction: none
1566 measured reflections
1405 independent reflections
1235 reflections with *I* > 2σ(*I*)
*R*_int_ = 0.053
3 standard reflections every 97 reflections intensity decay: none

#### Refinement

*R*[*F* ^2^ > 2σ(*F* ^2^)] = 0.034
*wR*(*F* ^2^) = 0.100
*S* = 0.95
1405 reflections
92 parameters
H-atom parameters constrained
Δρ_max_ = 0.41 e Å^−3^
Δρ_min_ = −0.38 e Å^−3^

### 

Data collection: *XSCANS* (Bruker, 1996 ▶); cell refinement: *XSCANS*; data reduction: *XSCANS*; program(s) used to solve structure: *SHELXS97* (Sheldrick, 2008 ▶); program(s) used to refine structure: *SHELXL97* (Sheldrick, 2008 ▶); molecular graphics: *SHELXTL* (Sheldrick, 2008 ▶); software used to prepare material for publication: *SHELXTL*.

## Supplementary Material

Crystal structure: contains datablocks I, global. DOI: 10.1107/S1600536809034606/pv2198sup1.cif

Structure factors: contains datablocks I. DOI: 10.1107/S1600536809034606/pv2198Isup2.hkl

Additional supplementary materials:  crystallographic information; 3D view; checkCIF report

## Figures and Tables

**Table 1:** Hydrogen-bond geometry (Å, °)

*D*—H⋯*A*	*D*—H	H⋯*A*	*D*⋯*A*	*D*—H⋯*A*
N3—H3⋯O2^i^	0.86	1.99	2.845 (2)	174

Symmetry code: (i) 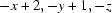.

**Fig. 1.**
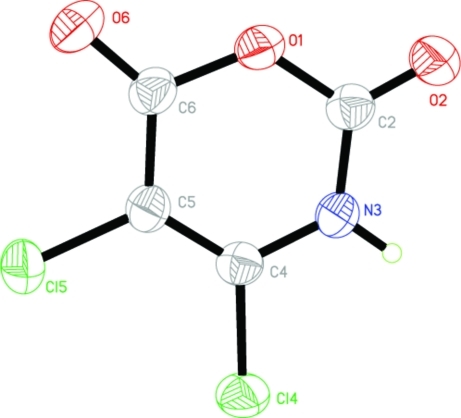
The molecular structure of the title compound, with atom labels and 50% probability displacement ellipsoids for non-H atoms.

**Fig. 2.**
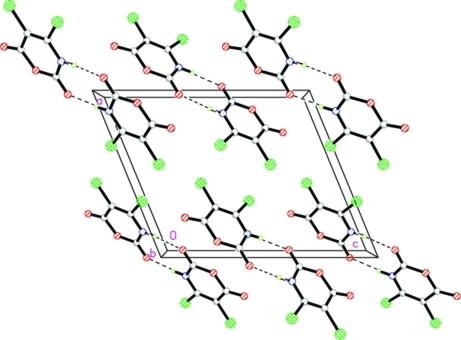
The packing of the title compound viewed along the b axis and showing the H-bonded dimer formed by inversion related molecules.

## supplementary crystallographic information

### Comment

The synthesis of derivatives of 3-oxauracil has previously been reported (Warren *et al.*, 1975) and an improved synthesis of the unsubstituted 3-oxauracil was reported by Rehberg & Glass (1995). The structure of the unsubstituted 3-oxauracil and its monohydrate have been reported (Copley *et al.*, 2005). Three derivatives of 3-oxauracil (4-methyl, 4-bromo, and 4,5-dichloro) have been prepared in our laboratory in route to the synthesis of 1-aza-1,3-butadienes. In this paper, we report the crystal structure of the title compound, (I).

Unlike the hydrogen bonding observed in 4-methyl derivative (Parrish, Leuschner *et al.*, 2009) resulting in staggered chains of molecules, in the crystal structure of of the title compound (Fig. 1), the molecules of (I) are held together by classical intermolecular hydrogen bonds of the type N—H···O resulting in dimeric units about inversion centers, forming eight membered ring systems which may be described in terms of graph set notation (Bernstein *et al.* 1994) as R_2_^2^(8) ring motif (details have been given in Table 1 and Figure 2). The molecular dimensions in (I) agree well with the corresponding bond distances and angles reported for the above mentioned structures and 4-boromo derivative of 3-oxauracil (Parrish, Tivitmahaisoon *et al.*, 2009).

### Experimental

Dichloromaleic anhydride (3,4-dichlorofuran-2,5-dione) and trimethylsilyl azide were treated analogously to the syntheses reported for the 4-methyl (Parrish, Leuschner *et al.*, 2009) and 4-bromo derivatives. Crystals of the title compound were grown from a solution of acetone at room temperature by slow evaporation.

### Refinement

Hydrogen atom bonded to N3 was calculated and refined using a riding model using the N—H distance 0.88 Å with *U*_iso_(H) = 1.2*U*_eq_(N).

### Crystal data

**Table:**

C_4_HCl_2_NO_3_	*F*(000) = 360
*M**_r_* = 181.96	*D*_x_ = 1.980 Mg m^−^^3^*D*_m_ = 1.92 Mg m^−^^3^*D*_m_ measured by floatation
Monoclinic, *P*2_1_/*c*	Mo *K*α radiation, λ = 0.71073 Å
Hall symbol: -P 2ybc	Cell parameters from 20 reflections
*a* = 10.2290 (16) Å	θ = 10–12.5°
*b* = 5.2549 (8) Å	µ = 1.00 mm^−^^1^
*c* = 12.2766 (16) Å	*T* = 293 K
β = 112.359 (11)°	Plates, colorless
*V* = 610.28 (16) Å^3^	0.38 × 0.33 × 0.15 mm
*Z* = 4	

### Data collection

**Table:**

Siemens R3m/V diffractometer	*R*_int_ = 0.053
Radiation source: fine-focus sealed tube	θ_max_ = 27.6°, θ_min_ = 2.2°
graphite	*h* = 0→13
θ–2θ scans	*k* = 0→6
1566 measured reflections	*l* = −15→14
1405 independent reflections	3 standard reflections every 97 reflections
1235 reflections with *I* > 2σ(*I*)	intensity decay: none

### Refinement

**Table:**

Refinement on *F*^2^	Secondary atom site location: difference Fourier map
Least-squares matrix: full	Hydrogen site location: inferred from neighbouring sites
*R*[*F*^2^ > 2σ(*F*^2^)] = 0.034	H-atom parameters constrained
*wR*(*F*^2^) = 0.100	*w* = 1/[σ^2^(*F*_o_^2^) + (0.0666*P*)^2^ + 0.3617*P*] where *P* = (*F*_o_^2^ + 2*F*_c_^2^)/3
*S* = 0.95	(Δ/σ)_max_ < 0.001
1405 reflections	Δρ_max_ = 0.41 e Å^−^^3^
92 parameters	Δρ_min_ = −0.38 e Å^−^^3^
0 restraints	Extinction correction: *SHELXL97* (Sheldrick, 2008), Fc^*^=kFc[1+0.001xFc^2^λ^3^/sin(2θ)]^-1/4^
Primary atom site location: structure-invariant direct methods	Extinction coefficient: 0.042 (5)

### Special details

**Table:**

Geometry. All e.s.d.'s (except the e.s.d. in the dihedral angle between two l.s. planes) are estimated using the full covariance matrix. The cell e.s.d.'s are taken into account individually in the estimation of e.s.d.'s in distances, angles and torsion angles; correlations between e.s.d.'s in cell parameters are only used when they are defined by crystal symmetry. An approximate (isotropic) treatment of cell e.s.d.'s is used for estimating e.s.d.'s involving l.s. planes.
Refinement. Refinement of *F*^2^ against ALL reflections. The weighted *R*-factor *wR* and goodness of fit *S* are based on *F*^2^, conventional *R*-factors *R* are based on *F*, with *F* set to zero for negative *F*^2^. The threshold expression of *F*^2^ > σ(*F*^2^) is used only for calculating *R*-factors(gt) *etc*. and is not relevant to the choice of reflections for refinement. *R*-factors based on *F*^2^ are statistically about twice as large as those based on *F*, and *R*- factors based on ALL data will be even larger.

### Fractional atomic coordinates and isotropic or equivalent isotropic displacement parameters (Å^2^)

**Table:**

	*x*	*y*	*z*	*U*_iso_*/*U*_eq_	
O1	0.89484 (14)	0.7547 (3)	0.20549 (12)	0.0405 (4)	
C2	0.9358 (2)	0.6588 (4)	0.12050 (17)	0.0362 (4)	
O2	1.03514 (16)	0.7544 (3)	0.10600 (14)	0.0474 (4)	
N3	0.86084 (17)	0.4586 (3)	0.05845 (14)	0.0363 (4)	
H3	0.8864	0.3892	0.0062	0.044*	
C4	0.74604 (19)	0.3625 (3)	0.07572 (15)	0.0325 (4)	
Cl4	0.66660 (6)	0.11274 (10)	−0.01234 (4)	0.0453 (2)	
C5	0.7009 (2)	0.4611 (4)	0.15609 (16)	0.0347 (4)	
Cl5	0.55557 (6)	0.35198 (11)	0.17764 (5)	0.0491 (2)	
C6	0.7780 (2)	0.6694 (4)	0.22914 (17)	0.0366 (4)	
O6	0.75456 (18)	0.7746 (3)	0.30575 (15)	0.0533 (4)	

### Atomic displacement parameters (Å^2^)

**Table:**

	*U*^11^	*U*^22^	*U*^33^	*U*^12^	*U*^13^	*U*^23^
O1	0.0479 (8)	0.0401 (8)	0.0369 (7)	−0.0084 (6)	0.0199 (6)	−0.0094 (6)
C2	0.0401 (10)	0.0363 (9)	0.0321 (9)	−0.0007 (8)	0.0138 (8)	0.0003 (7)
O2	0.0479 (8)	0.0484 (9)	0.0515 (9)	−0.0126 (7)	0.0251 (7)	−0.0079 (7)
N3	0.0405 (8)	0.0411 (9)	0.0317 (8)	−0.0050 (7)	0.0188 (6)	−0.0060 (7)
C4	0.0366 (9)	0.0333 (9)	0.0256 (8)	−0.0020 (7)	0.0097 (7)	0.0002 (7)
Cl4	0.0530 (3)	0.0453 (3)	0.0375 (3)	−0.0132 (2)	0.0172 (2)	−0.0125 (2)
C5	0.0387 (9)	0.0387 (10)	0.0286 (8)	−0.0019 (8)	0.0148 (7)	0.0005 (7)
Cl5	0.0526 (3)	0.0587 (4)	0.0464 (3)	−0.0136 (2)	0.0303 (3)	−0.0082 (2)
C6	0.0442 (10)	0.0362 (9)	0.0317 (9)	−0.0008 (8)	0.0171 (8)	−0.0007 (7)
O6	0.0691 (10)	0.0521 (9)	0.0484 (9)	−0.0073 (8)	0.0334 (8)	−0.0166 (7)

### Geometric parameters (Å, °)

**Table:**

O1—C2	1.360 (2)	C4—C5	1.342 (3)
O1—C6	1.406 (2)	C4—Cl4	1.698 (2)
C2—O2	1.206 (2)	C5—C6	1.444 (3)
C2—N3	1.353 (3)	C5—Cl5	1.706 (2)
N3—C4	1.367 (2)	C6—O6	1.192 (2)
N3—H3	0.8600		
			
C2—O1—C6	125.02 (15)	C5—C4—Cl4	123.46 (15)
O2—C2—N3	124.69 (18)	N3—C4—Cl4	114.72 (14)
O2—C2—O1	118.79 (18)	C4—C5—C6	119.33 (17)
N3—C2—O1	116.51 (16)	C4—C5—Cl5	123.23 (15)
C2—N3—C4	122.41 (16)	C6—C5—Cl5	117.44 (14)
C2—N3—H3	118.8	O6—C6—O1	117.20 (18)
C4—N3—H3	118.8	O6—C6—C5	127.99 (19)
C5—C4—N3	121.82 (17)	O1—C6—C5	114.81 (16)
			
C6—O1—C2—O2	177.41 (18)	N3—C4—C5—Cl5	178.26 (14)
C6—O1—C2—N3	−3.1 (3)	Cl4—C4—C5—Cl5	−1.5 (3)
O2—C2—N3—C4	−177.81 (19)	C2—O1—C6—O6	−179.15 (19)
O1—C2—N3—C4	2.7 (3)	C2—O1—C6—C5	1.0 (3)
C2—N3—C4—C5	−0.3 (3)	C4—C5—C6—O6	−178.2 (2)
C2—N3—C4—Cl4	179.47 (15)	Cl5—C5—C6—O6	1.5 (3)
N3—C4—C5—C6	−2.0 (3)	C4—C5—C6—O1	1.6 (3)
Cl4—C4—C5—C6	178.29 (14)	Cl5—C5—C6—O1	−178.62 (13)

### Hydrogen-bond geometry (Å, °)

**Table:**

*D*—H···*A*	*D*—H	H···*A*	*D*···*A*	*D*—H···*A*
N3—H3···O2^i^	0.86	1.99	2.845 (2)	174

Symmetry codes: (i) −*x*+2, −*y*+1, −*z*.
